# Facile Synthesized Cu–RGO and Ag–RGO Nanocomposites with Potential Biomedical Applications

**DOI:** 10.3390/nano12122096

**Published:** 2022-06-17

**Authors:** Diana Kichukova, Ivanka Spassova, Aneliya Kostadinova, Anna Staneva, Daniela Kovacheva

**Affiliations:** 1 Institute of General and Inorganic Chemistry, Bulgarian Academy of Sciences, 1113 Sofia, Bulgaria; diana123georgieva@gmail.com (D.K.); ispasova@svr.igic.bas.bg (I.S.); 2 Institute of Biophysics and Biomedical Engineering, Bulgarian Academy of Sciences, 1113 Sofia, Bulgaria; anik@bio21.bas.bg; 3 Faculty of Metallurgy and Materials Science, University of Chemical Technology and Metallurgy, 1756 Sofia, Bulgaria; ani_sta@mail.bg

**Keywords:** Cu–RGO, Ag–RGO, nanocomposites, facile synthesis, antibacterial, antifungal, cytotoxicity

## Abstract

In the present study, we report on the facile prepared nanocomposites of reduced graphene oxide RGO with Cu and Ag. The synthesis was performed through an environmentally friendly and easy method by simultaneous reduction in solutions containing Cu^2+^ or Ag^+^ and graphene oxide (GO) using zinc powder as a reducing agent in aqueous acidic media. The composites are characterized by powder X-ray diffraction, low-temperature nitrogen adsorption, X-ray photoelectron and FTIR and Raman spectroscopies, as well as Scanning and Transmission electron microscopies. The antibacterial activity of the composites was tested for *Staphylococcus aureus, Escherichia coli* and antifungal activity for *Candida albicans*. The cytotoxicity of the materials was studied towards two types of eukaryotic cells—MDCK II and A549 cell lines. The composites obtained consist of homogeneously distributed Cu and Ag nanoparticles on the surface of graphene sheets and manifest good antimicrobial activity and high cytotoxicity. The results clearly show that both metal–RGO composites can be successfully used as antimicrobial and anticancer agents.

## 1. Introduction

The use of materials with antibacterial and antifungal properties is widely presented in modern everyday life [1]. The aim is to stop or slow bacterial and fungal growth. Their use is absolutely necessary in hospitals to prevent the spread of pathogens among the staff, the patients and the visitors. Therefore, treatment with disinfecting agents on surfaces, tools, fabrics or packaging is required. The use of some powerful inorganic and organic disinfectants is limited due to their toxicity to the human body [2]. The use of antibiotics is not highly recommended because they can remain on the treated areas. Another problem associated with the use of antibiotics is that some bacterial strains become resistant to them. Resistant bacteria circulating in a hospital environment are particularly dangerous, as long-term use of antibiotics has led to the emergence of various defense mechanisms in bacteria [3].

Recently, due to its high specific surface area, the presence of a large number of surface functional groups and rapid electron transfer, graphene-based nanomaterials have been reported to have promising performance for adsorption of gases and remediation of water by removing pollutants from the environment. These materials are reported to be active towards various environmental pollutants and can be used in removal of dyes, organic contaminants, heavy metals and non-metals in water media. Another application is for the purification and separation of gases [4]. Control of the growth of microorganisms is important in the disinfection of water, filtration of air and for antimicrobial coatings on surfaces. The antimicrobial activity of graphene is attributed to two mechanisms: by physical rupture of the cell’s membranes and by oxidative stress caused by the reactive oxygen species or by electron transfer [5,6]. The antimicrobial properties of graphene are of considerable interest because the mechanisms of the cytotoxicity do not require high energy consumption or replenishment of biocidal agents. It is well known that graphene oxide (GO) and reduced graphene oxide (RGO) have cytotoxicity and are biocompatible materials that exhibit broad activity against bacterial pathogens [7,8]. For its antimicrobial action, graphene is used in the packaging of foodstuffs or to cover biomedical equipment where bacterial multiplication on the surface is undesirable. It was shown that with modification of graphene by metal or metal oxide nanoparticles, a synergic effect occurs and the antibacterial action is more effective due to the fine structure, new surface characteristics and durability of the composite obtained [9]. Polymer–graphene based composites also show prospective antibacterial properties [10].

The biocidal properties of silver have been recognized since ancient times and used for this purpose throughout human history. Silver is either applied directly, dissolved in water or anchored to inorganic substances from which it can be released [11]. It is known that silver ions show low toxicity to human or animal cells but are toxic to bacteria, which do not develop resistance to silver. However, the wide application of silver as a disinfecting agent is complicated due to its high cost. Copper is also known to be active against bacteria and fungi [12,13] and is widely used in materials for such purposes. The traditional use of silver and copper-containing solutions causes problems with both the correct dosage and the fact that the treatment has a short-term effect. Metal nanoparticles would release ions in small doses and for a longer period, thus, antibacterial treatment would be more reliable [14]. Metal nanoparticles can be successfully stabilized by application on a suitable carrier. Therefore, combining the properties of RGO, copper and silver in nanoscale metal–RGO composites would be promising for the preparation of materials with antibacterial and antifungal activities [15]. They could be used as an attractive way to overcome the resistance of pathogenic microorganisms to commonly used antibacterial products. Most of the recent available papers focus mainly on the antibacterial effects of the materials, but only a few studies have been dedicated to antifungal properties.

Recently, it was published that the metal assisted GO reduction proceeds via the generated gaseous H_2_ [16,17]. The reducing potential of Mg is highest, but because of the fast kinetics of the evolved gaseous hydrogen, its application is not common. More suitable metals with high reducing potential for such reactions are Al and Zn because of their abundance, safety and price [18,19].

The aim of this study was to propose an easy and environmentally friendly method for the synthesis of Cu–RGO or Ag–RGO composites and to study their behavior towards the bacteria *Staphylococcus aureus* and *Escherichia coli* and the fungus *Candida albicans* as well as their cytotoxicity towards some eukaryotic cells.

## 2. Materials and Methods

### 2.1. Preparation of Graphene Oxide (GO)

Graphene oxide was obtained by chemical exfoliation of natural graphite powder (Sigma-Aldrich, Saint Louis, MO, USA99% carbon, 50 meshes) by the Tour method [20]. Prior to synthesis, graphite was mechanically milled for 1 h (rotation speed 5000 rpm) in a planetary ball mill. The grinding vessel and balls were made of ZrO_2_. For oxidation, 1 g of ground graphite powder was mixed with 40 mL of H_2_SO_4_ and 5 mL of H_3_PO_4_. The suspension was stirred for 30 min vigorously in an ice bath. Then, 6 g of KMnO_4_ was added dropwise in order to avoid the increase in the reaction temperature. After stirring, 100 mL of distilled water were added. The resulting mixture was sonicated for 15 min using a Sonix ultrasonic processor (20 KHz, 750 W) and left to stand for 3 h. Further, a solution of 50 mL of 30% H_2_O_2_ in 80 mL of water was added in small drops. After 1 h of stirring, the mixture was washed with 1M HCl solution and with deionized water to pH ≈ 6. The obtained graphene oxide was dried at 90 °C for 12 h.

### 2.2. Preparation of Composites Cu–RGO, Ag–RGO and RGO

The composites were obtained by simultaneous reduction in GO and metal salts to RGO decorated with corresponding metals. A 100 mL suspension containing 0.3 g GO was placed, along with 0.1 g powdered metal Zn, in a laboratory dish followed by addition of 10 mL of concentrated HCl. During the reaction of HCl with Zn, the released hydrogen reduced the GO to RGO. The quantities of the metal salts were 0.04 g Cu(CH_3_COO)_2_·4H_2_O or 0.02 g AgNO_3_. The metal salts were added to the reaction mixture with the last portion of HCl. The reduction in metal salts is rapid, taking about 5 min. After the reduction, the samples were washed to pH ≈ 6 and dried at 90 °C for 12 h. The as-obtained samples were denoted as Cu–RGO and Ag–RGO. For the sake of comparison, pure RGO was additionally prepared by the described reduction technique and denoted as RGO. The reduction mechanism of the synthesis procedure could be summarized as follows: Initially, due to the oxidation of Zn powder, it produces electrons and, simultaneously, Cu^2+^ (Ag^+^) ions and GO receive electrons and become reduced. Then, the generated electrons also react with proton (H^+^) and produce gaseous hydrogen.

### 2.3. Sample Characterization

Powder XRD was performed on a Bruker D8 Advance powder diffractometer with Cu Kα radiation (λ = 1.5406 Å) and a LynxEye detector in order to investigate the phase composition and crystallite size of the materials and composites obtained in this study. The experiments were performed from 10 to 90° 2θ with a step 0.03° 2θ and counting time 57 s/step. The rotation speed of the sample was 15 rpm. The phase composition was determined by Diffracplus EVA and the ICDD-PDF2 (2019) database. The mean coherent domain sizes (LVol-IB) were determined by Topas-version 4.2 software (Karlsruhe, Germany).

The morphology of the samples was studied by Scanning Electron Microscopy (SEM) on a JEOL-JSM-6390 (Tokyo, Japan) equipped with EDS (Oxford Instruments, UK).

Transmission Electron Micrographs (TEM) were taken on a TEM JEOL 2100 at 200 kV, using samples suspended onto a standard C/Cu grid.

The low temperature nitrogen adsorption (77.4K) was performed on a NOVA 1200e instrument (Quantachrome, Boynton Beach, FL, USA). Before the measurements, the samples were treated at 80 °C for 16 h in a vacuum. The specific surface areas (S_BET_) were calculated using the BET equation; the total pore volumes (V_t_) and average pore diameters (D_av_) were estimated at p/p_o_ close to the end of the isotherm.

The FTIR spectra of samples were collected by a Thermo Scientific Nicolet iS5 Fourier-transform infrared instrument. The resolution was 2 cm^−1^ with 64 scans accumulating in the range of 4000–400 cm^−1^.

The Raman spectra were registered using a triple multichannel spectrometer, Microdil 28 (Dilor), with a He-Ne laser with a wavelength of 633 nm and optical microscope. The scattered light was collected in backward scattering geometry.

The X-ray photoelectron spectroscopy (XPS) analyses were carried out in the ESCALAB-Mk II (VG Scientific, Manchester, UK) electron spectrometer with Al Kα radiation (hν = 1486.6 eV).

### 2.4. Antimicrobial Tests

The antimicrobial tests were performed according to the following procedure: a 4 mg sample was placed in 2 mL saline, and 50 µL of these suspensions were added to 980 µL of microorganism suspension with a concentration ≈10^7^ cells/mL. In such mixture, the amount of the composite is ≈100 µg/mL. These incubation mixtures were vigorously shaken at room temperature and test samples were taken from them at 0 h, 3 h and 24 h. The test samples were diluted ten times, inoculated on soy casein agar with addition of yeast extract and incubated at 37 °C. The procedure is described in [21]. The number of the colony forming units was recorded after 24 h. The control (without active material) sample was also tested.

The microorganisms tested were: *Staphylococcus aureus* NBIMCC 3359 (corresponding to ATCC 6538); *Escherichia coli* NBIMCC 3397 (ATCC 8739); *Candida albicans* NBIMCC 74(ATCC 10231), where NBIMCC is the National Bank for Industrial Microorganisms and Cell Culture and ATCC is the American Type Culture Collection.

### 2.5. Cytotoxicity Tests

For the cytotoxicity tests, the pure RGO and the metal–RGO composites with concentrations 100 µg/mL were placed in 24 wells. The cells tested were seeded with a concentration of 1 × 10^5^ cells/mL. The cytotoxicity was determined after 24 h using the crystal violet test and the morphology of the cells was inspected microscopically. For this purpose, the residual cell monolayer was washed with phosphate-buffered saline fixed with 4% paraformaldehyde for 15 min, followed by washing with water. Then, 200 µL of 0.1% crystal-violet solutions were added to every well. The incubation at room temperature was set for 20 min and was followed by washing with water. The protein-bound dye connected to the cell number was extracted with 200 µL 10% CH_3_COOH [22]. The optical densities were determined using EPOCH UV/VIS Spectrometer at 570 nm, and the percentage of vital cells was calculated. All experiments were triple repeated and the average value was calculated. The microscope pictures were taken with a digital camera DV-130, XDS-2A microscope (Guangxi, China).

The cytotoxicity of the materials was studied towards two types of eukaryotic cells: kidney epithelial cell line—MDCK II and alveolar sarcoma cell line—A549, provided by the National Bank for Industrial Microorganisms and Cell Cultures (NBIMCC, Sofia, Bulgaria). The eukaryotic cells were kept at standard conditions (humidified atmosphere, 5% CO_2_, 37 °C) in Dulbecco′s Modified Eagle′s Medium D6429 (Merck, Darmstadt, Germany) with 10% Fetal Bovine Serum (FBS) (BioWhittaker TM, Lonza, Swiss) and 1% (*v*/*v*) antibiotic-antimycotic solution (penicillin 100 U/mL, streptomycin 100 µg/mL and amphotericin B 0.25 µg/mL (BioWhittaker TM, Lonza, Swiss)).

## 3. Results and Discussion

### 3.1. Powder XRD Analysis

**Figure 1:**
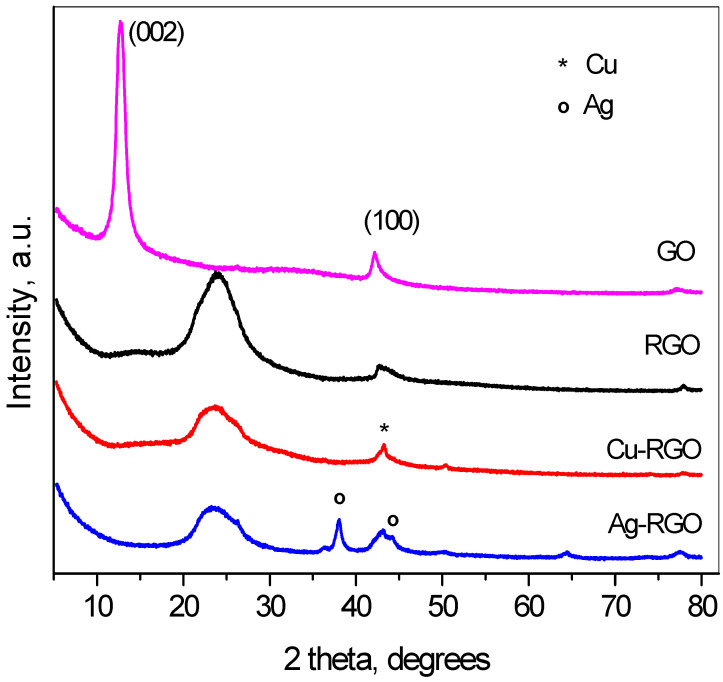
Powder X-ray diffraction patterns of GO, RGO and the composites Cu–RGO and Ag–RGO. The symbols denote: *—metallic copper, ◦—metallic silver.

In Figure 1, the powder diffraction patterns of GO, RGO and the composites can be seen. The graphene oxide pattern consists of two pronounced peaks—first in the range of 10–12° 2θ, that corresponds to the interplanar distances 7.5 to 8.5 Å, characteristic for the (002) distances, and the second peak at ~42.5° 2θ, originating from the (100) distances in the carbon layers.

This pattern confirms the oxidation of the initial graphite. The complete reduction in GO could be seen from the pattern of RGO with the shift of (002) peak to about 25° 2θ, where the interplanar distance is 3.2–3.4 Å, indicating the successful functional groups’ removal. The reduction in GO to RGO proceeds simultaneously to the reduction in the metal ions in the solution, and the neutral metal atoms settle on the surface of the graphene sheets. Thus, the metal phases are formed and the corresponding reflections of metals are seen on the XRD patterns. The width of the (002) peak of graphene increases after reduction, as the reduction process leads to the tearing of the sheets and to lowering the number of stacked graphene sheets. The XRD patterns of composites with copper and silver present peaks evidencing the formation of metallic copper and silver. Traces of Cu_2_O are also detected, indicating partial surface oxidation of the Cu nanoparticles. The analyses of diffraction line broadening [23] resulted in the crystallite sizes as follows: GO—8 nm, RGO—3 nm, Cu in Cu–RGO—11 nm and Ag in Ag–RGO—13 nm.

### 3.2. Scanning Electron Microscopy

**Figure 2:**
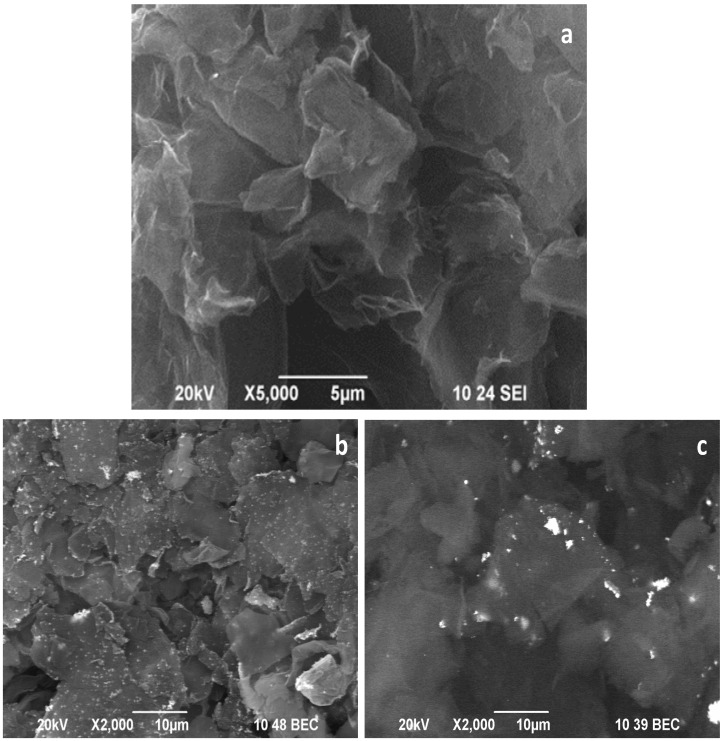
SEM images of: (**a**) RGO in SEI mode, (**b**) Cu–RGO and (**c**) Ag–RGO in BEC mode.

The SEM image in Figure 2a shows that the applied procedure of oxidation and reduction leads to the preparation of RGO material with well-shuffled graphene sheets originating from successful exfoliation of graphite precursor. The morphology of the obtained RGO is similar to that obtained by other procedures [24]. Figure 2b,c represents the composites’ morphology of Cu–RGO and Ag–RGO, respectively. An excellent distribution of copper and silver nanoparticles can be seen on the surface of the graphene sheets as the metal particles (bright spots in the images) are in good contrast to the background of RGO. One can note that the silver nanoparticles aggregate to form larger clusters that tend to be situated at the edges of graphene sheets.

### 3.3. Transmission Electron Microscopy

**Figure 3:**
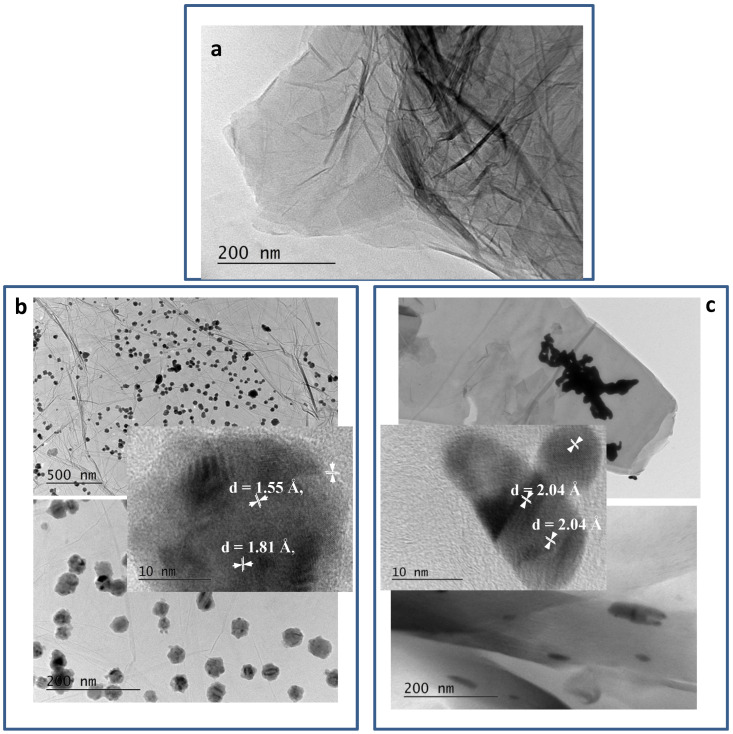
TEM images of: (**a**) RGO, (**b**) Cu–RGO/Cu, (**c**) Ag–RGO.

Figure 3 shows the bright field micrographs of RGO and metal–RGO composites. In Figure 3a, the common sheet-like morphology [25] of the pure RGO prepared by zinc-assisted reduction procedure is shown. The sheets are large and plicate. Figure 3b,c shows the typical views at different magnifications as well as HRTEM images of the two metal–RGO composites. A clear difference is evident. For Cu–RGO composites, the Cu particles are almost spherical and are arrayed evenly on the surface of the graphene sheets. They do not aggregate and stand isolated from each other on the surface, covering a large area of the sheets. On the HRTEM image, one could see the corresponding interplanar distances (200) of Cu° (d = 1.81 Å) and (220) of Cu_2_O (d = 1.55 Å) in the HRTEM of Cu–RGO. The distances mentioned in the HRTEM image Ag–RGO correspond to the interplanar distances (200) of Ag° (d = 2.04 Å). The presence of Cu_2_O is seen on the outer surface of the particle due to the partial oxidation in air. On the other hand, the Ag nanoparticles having elongated shapes form relatively large aggregates, located predominantly at the graphene sheets’ edges.

### 3.4. N_2_-Physisorption

**Figure 4:**
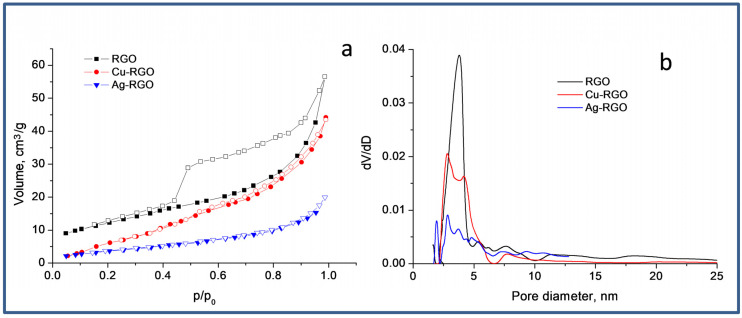
Nitrogen adsorption–desorption isotherms (**a**) and pore-size distributions (**b**).

Low-temperature nitrogen adsorption–desorption measurements were made in order to define the surface and the porous structure of RGO and the composites. The nitrogen adsorption–desorption isotherms of the prepared samples are shown in Figure 4a, and the pore-size distributions are shown in Figure 4b. The BET specific surface areas (S_BET_), total pore volumes (V_t_) and average pore sizes D_av_ are summarized in Table 1.

The studied samples have isotherms of mixed type II–IV with H3 hysteresis at p/p_0_ from 0.4 to 1.0 in range, which is an indication of the formation of mesopores between the slit-shaped pores [26]. The RGO exhibits a Brunauer−Emmett−Teller (BET) specific surface area of 45 m^2^g^−1^. From the texture parameters of Cu–RGO and Ag–RGO, it could be seen that the formation of the composites does not change the texture and morphology of the RGO significantly, but only lowers the specific surface area and pore volumes due to partial filling or blocking of the pores. However, copper particles are more homogeneously distributed on the RGO sheets than the silver particles.

This confirms the observations from SEM and TEM that silver tends to form bigger aggregates than copper. The pore size distributions made by the NLDFT method display that most of the pore volume of all samples is in the 2.5−10 nm range, with a main peak centered at ~3.6 nm, indicating the strong mesoporosity of the pure RGO and the composites.

### 3.5. FTIR Spectroscopy

**Figure 5:**
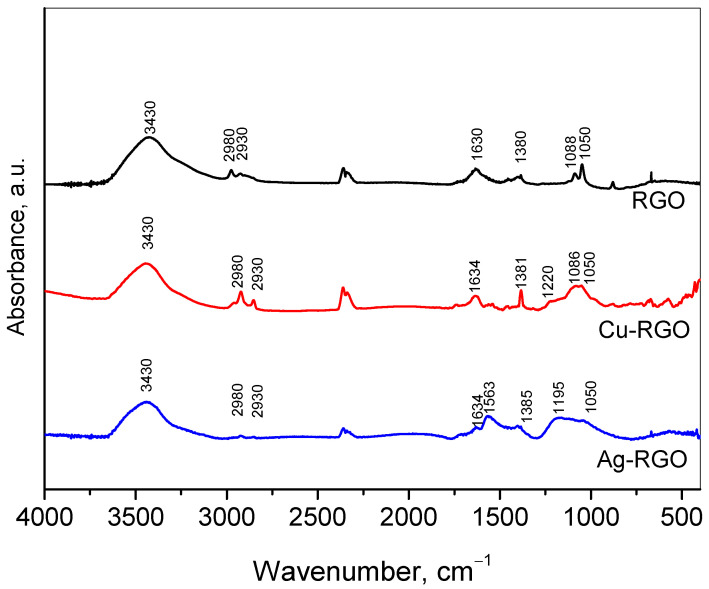
FTIR spectra of RGO, Cu–RGO/Cu and Ag–RGO.

The FTIR spectra of RGO, Cu–RGO and Ag–RGO are shown in Figure 5. In the FTIR spectra of RGO and the composites, a broad peak centered at about 3430 cm^−1^ is assigned to the O−H stretching vibration of H_2_O molecules, being less intensive for the composites; this suggests the partial removal of the surface adsorbed water.

The strong peak at 1630 cm^−1^ assigned to C=C stretching, along with the double peaks at 2980 and 2830 cm^−1^, indicates a preservation of the carbon basal planes in RGO [27]. The peaks at 1380 cm^−1^, 1088 cm^−1^ and 1050 cm^−1^ could be assigned to C−OH bending, alkoxy C−O stretching and C−OH stretching, respectively [28,29]. In the spectra of the composites Cu–RGO and Ag–RGO along with the upper mention bands, slight differences could be seen. The bands at about 1220 cm^−1^ for Cu–RGO and 1195 cm^−1^ for Ag–RGO could be connected to the C–O–C vibrations [30,31].

### 3.6. Raman Spectroscopy

**Figure 6:**
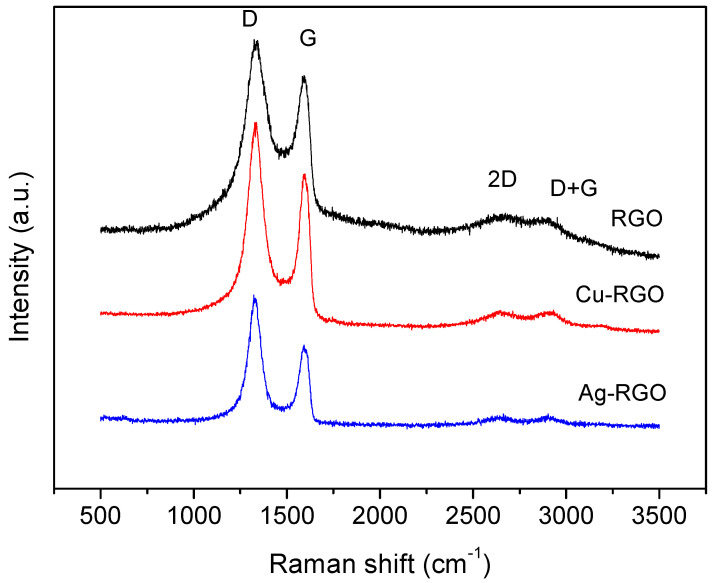
Raman spectra of RGO and the composites Cu–RGO and Ag–RGO. Letters denote major grapheme Raman modes: D—band, G—band, 2D—obertone, D + G—combinational band.

Raman spectra contain useful information for the characterization of graphene materials, providing insights into the structural changes, type of defects and the metal’s influence. In Figure 6, the more pronounced Raman bands of RGO, Cu–RGO and Ag–RGO are presented.

The two most intensive bands are in the range from 1100 to 1700 cm^−1^. The G-band is characteristic for graphite and graphene, representing the interplanar C–C stretching mode of the carbon rings (configuration sp^2^). The intensity of the G band relates to the thickness of the stacked graphene sheets. The width of the G-band reflects the deformation and strain in the graphene sheets. The D-band represents a ring breathing mode from the carbon rings and arises from the presence of disorder, edges and defects of the graphene sheets. The band is usually very weak in graphite and strong in amorphous carbon. The intensity of the D-band is a measure for the degree of the defects in the sample. In the present study, the position of the G band is at 1597 cm^−1^ for RGO and the composite samples, while the position of the D-band is 1340 cm^−1^, 1332 cm^−1^ and 1327 cm^−1^ for RGO, Cu–RGO and Ag–RGO, respectively. The D-band shift suggests that metal nanoparticles affect the electronic structure of the graphene sheets via p-doping effects [32,33].

A more accurate estimation of the disorder degree is the ratio of the intensities of D and G bands (I_D_/I_G_). In our case, the I_D_/I_G_ is 1.21 for the RGO, which confirms a good quality of the material prepared by the proposed method [20]. For the composite materials, the calculated I_D_/I_G_ values are 1.33 and 1.56 for Cu–RGO and Ag–RGO, respectively. These values indicate the high level of defects presented in the composites, related to simultaneous formation of the RGO and metal nanoparticles. The effect is more pronounced for the Ag–RGO composite, which confirms the observations from SEM, TEM and N_2_ physisorption.

### 3.7. X-ray Photoelectron Spectroscopy

**Figure 7:**
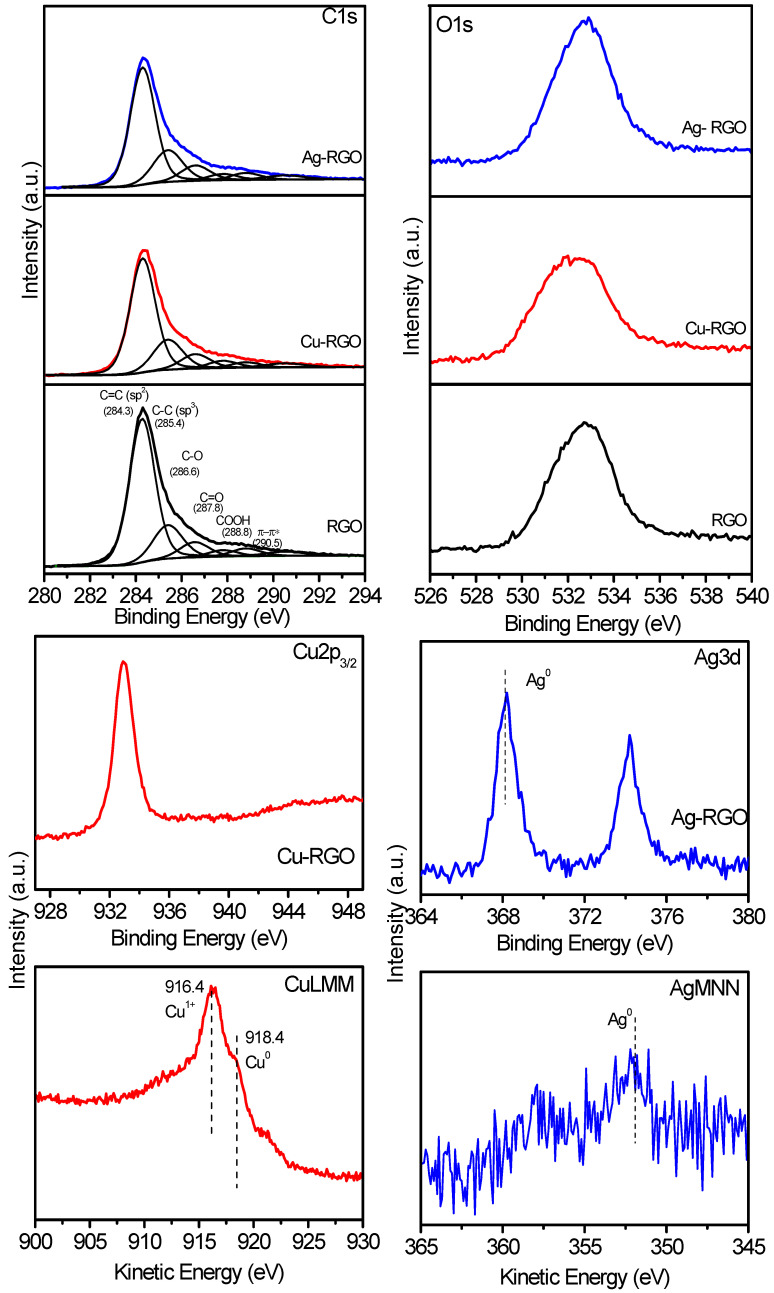
XPS spectra of C 1s, O 1s, Cu 2p_3/2_, Ag 3d, Cu LMM and Ag MNN for RGO and the composites Cu–RGO and Ag–RGO.

XPS measurements were performed to investigate the chemical state of C, O, Cu and Ag in the RGO and the composites Cu–RGO and Ag–RGO (Figure 7).

The C1s XPS peak is asymmetrical for all samples and represents a superposition of lines, corresponding to different C-bonds as follows: 284.3 eV for C=C (sp^2^), 285.4 eV for C−C (sp^3^), 286.6 eV for C−O, 287.8 eV for C=O, 288.8 eV for COOH and 290.5 eV for π−π*. All these bonds are typical for the RGO materials. The presence of metal nanoparticles in the composites does not substantially affect the bonds formed by carbon atoms.

The XPS spectra of O1s core levels are similar in position and shape for the pure RGO and Ag–RGO composite. For the Cu–RGO sample, a slight broadening of the O1s peak is observed due to the presence of Cu−O bonds originating from the oxidized surface of the Cu nanoparticles.

Regarding the Cu 2p_3/2_ spectrum, binding energies greater than 934.4 eV are attributed to Cu^2+^ in CuO, and binding energy of ~932.7 eV is considered for Cu^+^ in Cu_2_O [34]. The position of the line for Cu–RGO binding energy of 932.8 eV and the absence of a satellite could be related to copper in the low oxidation state. This is confirmed by the Auger spectrum (Cu LMM). The kinetic energies of Cu_2_O, CuO and Cu are reported as 916.4 eV, 917.7 eV and 918.6 eV, respectively [35], and it is evident that the kinetic energy for Cu–RGO composite corresponds to mixed Cu° and Cu^+^ states, confirming the observations in HRTEM.

The Ag 3d spectrum for Ag–RGO evidences the formation of Ag° particles—the binding energy in the Ag3d_5/2_ peak is 368.3 eV [36]. This is supported by the Auger Ag MNN spectrum, where the kinetic energy is 351 eV. The observations from XPS confirm the data of XRD and HRTEM.

### 3.8. Antimicrobial Activity

**Figure 8:**
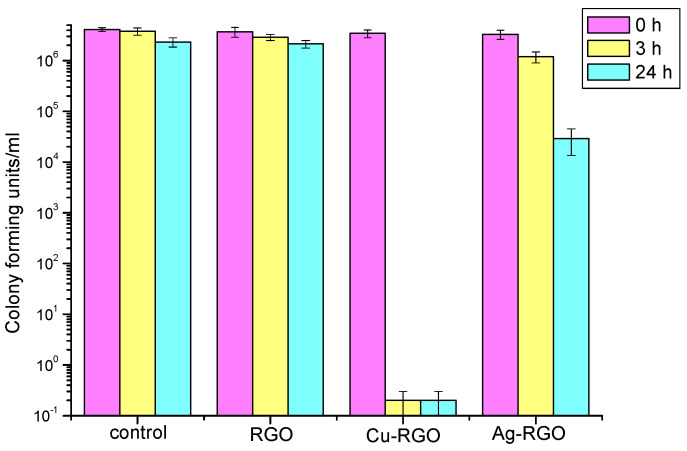
Survival of *Staphylococcus aureus* bacteria after 0, 3 and 24 h treatment with RGO and metal–RGO composites.

**Figure 9:**
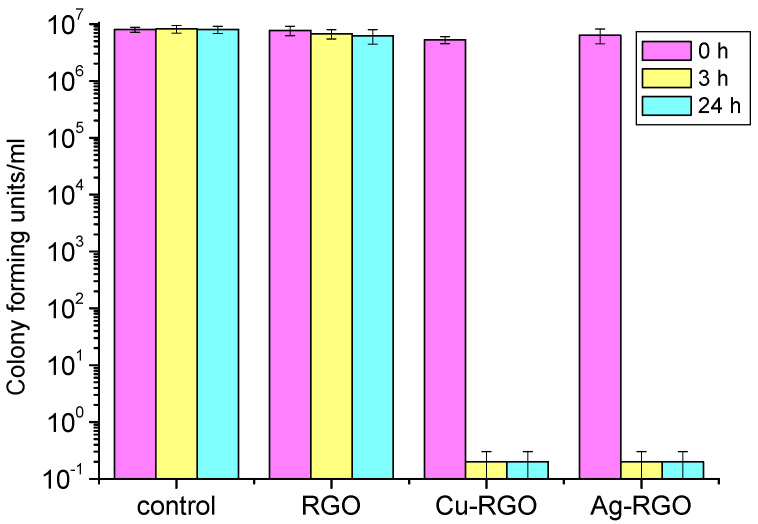
Survival of *Escherichia coli* bacteria after 0, 3 and 24 h treatment with RGO and metal–RGO composites.

**Figure 10:**
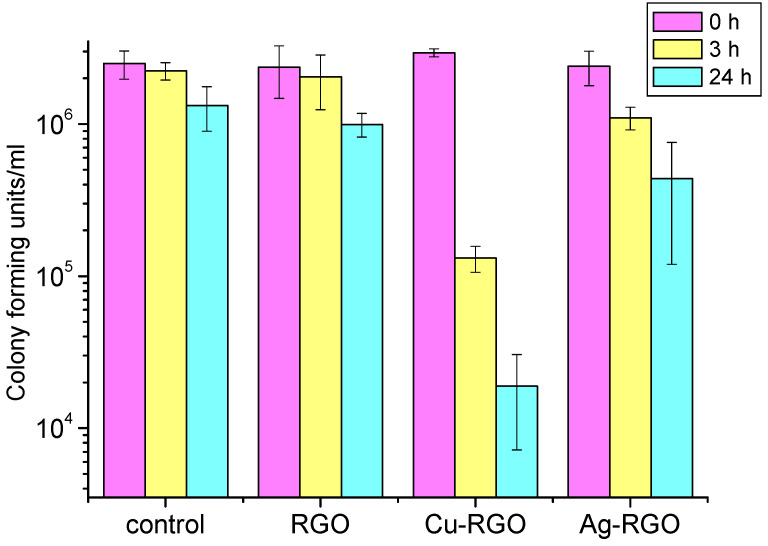
Survival of *Candida albicans* fungi after 0, 3 and 24 h treatment with RGO and metal–RGO composites.

The antibacterial effect of RGO and the metal–RGO composites was studied by measuring of the inhibition of the growth of the representatives of gram-positive (*Staphylococcus aureus*) and gram-negative (*Escherichia coli*) bacteria and fungus (*Candida albicans*). A control experiment without active material is used for comparison. The results are presented in Figure 8, Figure 9 and Figure 10, respectively.

For the treatment of *Staphylococcus aureus* (Figure 8), the antibacterial activity of pure RGO is negligible as the effect of inhibition is almost zero, being the same as that of the control for the entire time. On the other hand, visible inhibition effects are exhibited by treatment with metal–RGO composites. The Ag–RGO mildly suppresses the growth of these bacteria resulting in a reduction in the number of surviving bacteria by three orders of magnitude within 24 h. A huge effect is observed while treating the colony of *Staphylococcus aureus* by the Cu–RGO composite. One could see that the inhibition is significant even after 3 h. The suppression of bacterial growth after 3 h (24 h) is by eight orders of magnitude, leaving the incubation media practically free of bacteria.

For the treatment of *Escherichia coli* (Figure 9), both Ag–RGO and Cu–RGO composites show excellent inhibition properties within a short time (3 h). The suppression effect is almost the same for Ag–RGO and Cu–RGO and results in complete bacteria elimination. The pure RGO does not show any antibacterial activity.

In the case of treatment of *Candida albicans* fungi (Figure 10), the RGO itself shows a slight inhibition effect at the 24th hour. This observation confirms the results published by other authors [37,38]. The inhibition effect of the composites is more pronounced. For the Ag–RGO, the reduction in the number of surviving fungi is about 1.5 orders of magnitude after 24 h. The most active against *Candida albicans* is the Cu–RGO composite, where the colony forming units decrease with 2.5 orders of magnitude after 24 h. It should be mentioned that significant inhibition is observed even after 3 h of treatment with this composite.

However, the antifungal activity of the studied materials is lower than the antibacterial ones. Moreover, the activity against gram-negative *Escherichia coli* is much higher than against gram-positive *Staphylococcus aureus*. Such difference could be due to different patterns of the cell walls of the pathogens under study, as the cell walls of the gram-positive bacteria have a thickness of 20–80 nm and the gram-negative bacteria cell walls have a thickness of <10 nm [39]. Thus, one could assume different mechanisms of elimination and growth inhibition of the two bacteria. The bactericidal use of RGO is found to be limited because of graphene sheets’ stacking by van der Waals forces. In this case, only physical damages on the cell walls can be expected. The use of metal/metal oxide-RGO composites provides a large number of active species on the graphene surface and opens up other mechanisms for cell inhibition by chemical modes of action.

Such interaction is related to the oxidative stress caused by charge transfer and generation of reactive oxygen species. The oxidative stress is considered, by some authors, to be the major reason for bacteria elimination [40], while some others propose “capturing-killing process” elimination [41]. In the present case, the Cu–RGO composite appears to be the most active against all pathogens studied. The reasons for these results could relate not only to the bactericidal and antifungal properties of the copper itself (copper ions) but also to the specific morphology of the prepared composite. For the Cu–RGO composite, copper particles are small and well dispersed on the surface of the graphene sheets, ensuring maximal contact with the pathogen cells. The specific composite morphology also enables controlled slow-release of the metal ions.

### 3.9. Cytotoxicity

The investigation of eukaryotic cells was conducted with epithelial alveolar cell line A549, a high metastatic, and MDCK II epithelial kidney cell line, a low metastatic.

**Figure 11:**
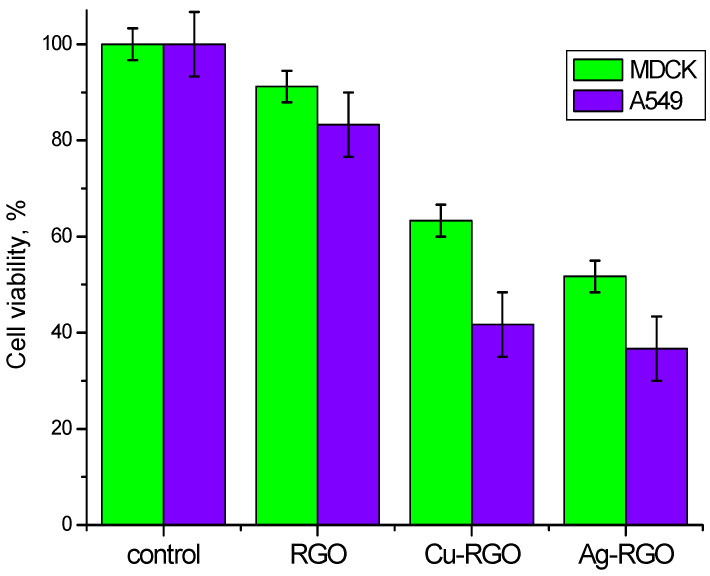
Cell viability after 24 h treatment with RGO and metal–RGO composites.

The spectrophotometric results, shown in Figure 11, reveal that the highest cell viability (above 80%) is observed for both cell lines incubated with pure RGO. Hence, pure RGO is assumed to be biocompatible and not to be cytotoxic (does not affect cells).

Different trends are observed for Cu–RGO and Ag–RGO nanocomposites. The treatment with both nanocomposites leads to a specific cell response of the two selected cell lines. For low metastatic MDCK II, the observed cell survival is higher than for the high metastatic A549. For both cell lines, the viability values are slightly higher for Cu–RGO treated cells compared to Ag–RGO treated cells. In general, both composites show significant reduction in the cells’ survival and can be successfully used as anticancer drugs.

**Figure 12:**
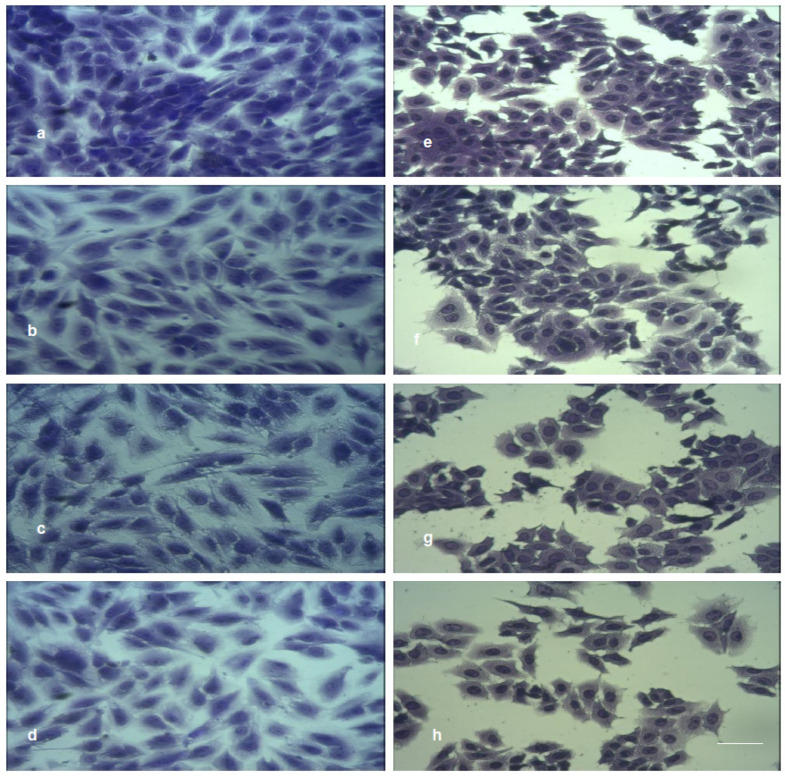
Cell morphology of MDCK II and A549 cell line on RGO and metal–RGO composites: (**a**) control MDCK II cells, (**b**) RGO, (**c**) Cu–RGO, (**d**) Ag–RGO; (**e**) control A549 cells, (**f**) RGO, (**g**) Cu–RGO, (**h**) Ag–RGO. Magnification 200×.

Figure 12 shows the initial cell morphology of MDCK II (a) and A549 (e) and morphology changes upon treatment with RGO (b,f), Cu–RGO (c,g) and Ag–RGO (d,h).

A549 cells exhibit a quick response to the treatment expressed by disturbance of cell–cell contacts and reduction in the cell number. Moreover, the cells forming smaller groups become more rounded.

The response of MDCK II cells upon treatment with pure RGO and with both nanocomposites is less pronounced due to the desmosomal cell contacts. The different cell response of the two lines is related to the specific metabolism of the two kinds of cells and their different shapes.

## 4. Conclusions

A simple, facile and straightforward approach for the preparation of metal–RGO nanocomposites is proposed. By this method, Cu–RGO and Ag–RGO materials with good antimicrobial activity and high cytotoxicity are obtained. A huge antimicrobial effect is observed via the Cu–RGO—the suppression of bacterial growth of *Staphylococcus aureus* and *Escherichia coli* is by 8 orders of magnitude (almost complete), and the inhibition effect against *Candida albicans* is about 2.5 orders of magnitude. The Ag–RGO antimicrobial effect is milder against *Staphylococcus aureus* and *Candida albicans* and the same as Cu–RGO against *Escherichia coli.* Both composites show high cytotoxicity, reducing the number of cells by approximately 40% for MDCK II and 60% for A549.

These biological features could be related to the specific phase composition and distribution as well as the materials’ morphology. The results for antibacterial and antifungal activities and cytotoxicity clearly show that both metal–RGO composites can be successfully used as antimicrobial and anticancer agents.

## Figures and Tables

**Table 1:** Texture parameters—specific surface area, total pore volume, average pore diameter.

Sample	S_BET_ [m^2^g^−1^]	V_t_ [cm^3^g^−1^]	D_av_ [nm]
RGO	45	0.09	8.0
Cu–RGO	36	0.06	6.8
Ag–RGO	17	0.04	9.2

## Data Availability

Not applicable.
